# *CX-ASAP*: a high-throughput tool for the serial refinement and analysis of crystallographic data collected under varying conditions

**DOI:** 10.1107/S1600576723000298

**Published:** 2023-02-28

**Authors:** Amy J. Thompson, Kate M. L. Smith, Jack K. Clegg, Jason R. Price

**Affiliations:** a School of Chemistry and Molecular Biosciences, The University of Queensland, St Lucia, Queensland 4072, Australia; b Swiss Light Source, Paul Scherrer Institute, Villigen PSI 5232, Switzerland; c Australian Synchrotron, ANSTO, Melbourne, 800 Blackburn Road, Clayton, Victoria 3168, Australia; DESY, Hamburg, Germany

**Keywords:** serial diffraction, serial refinement, computer programs, *CX-ASAP*

## Abstract

*CX-ASAP* is a new open-source software project designed to greatly reduce the time required to analyse crystallographic data collected under varying conditions. Scripted in Python3, *CX-ASAP* can automatically refine, finalize and analyse data collections with wide-ranging temperatures, pressures *etc*. This is achieved using a reference structure, allowing for quick identification of problems, phase changes and even model comparison. The modular design means that new features and customized scripts can be easily added, tailoring the capabilities to the specific needs of the user. It is envisioned that *CX-ASAP* will help to close the growing gap between fast collection times and slow data finalization.

## Abstract 2

*CX-ASAP* is a new open-source software project designed to greatly reduce the time required to analyse crystallographic data collected under varying conditions. This software will allow the rapid refinement and finalization of dynamic crystallographic experiments.

## 1. Introduction

Recent developments in X-ray detector and source technology are causing a step change in the practice of crystallography both at major facilities and in the home laboratory. The time required to collect a structure has reduced from hours/days to seconds/minutes, allowing for the rapid collection of data sets under varying conditions such as temperature or pressure. Though the data collection time is traditionally the slowest step of the crystallographic process (assuming samples are in hand), the advances in technology have moved the bottleneck to post-collection data processing and analysis.

It is now possible to collect more data than we can reasonably handle. For example, with robotic sample mounting it is common for synchrotron beamlines to generate more than 400 crystal structures in an 8 h shift, and single structures in as little as 1 s. There is a strong need for further software tools to assist with post-collection processes.

The challenges for large data sets in crystallography (Helliwell, 2019 ▸) and the necessity of aligning crystallographic data with the FAIR (findable, accessible, interoperable and reusable; Wilkinson *et al.*, 2016 ▸) and FACT (fairness, accuracy, confidentiality and transparency; van der Aalst *et al.*, 2017 ▸) principles are being increasingly recognized. These principles revolve around making data accessible, reusable and transparent, allowing for other researchers to easily validate findings. For instance, accessible diffraction images for published data sets would allow for external structure validation. The same ideals apply to crystallographic software. There is no need for everyone to write their own customized code to be a ‘black-box’ because collaborative open-source projects align better with these principles. In this article, we introduce a new open-source project with the aim of automating the serial refinement of crystallographic experiments.

### 1.1. Dynamic crystallographic experiments

The study of dynamic crystal systems is critical to understanding their properties. Many materials in crystalline form experience a structural change on the application of external stimuli such as temperature (Zuluaga *et al.*, 2020 ▸), pressure (Moggach *et al.*, 2009 ▸), light (Bushuyev *et al.*, 2014 ▸), force (Thompson, Worthy *et al.*, 2021 ▸) and more. Experiments investigating such changes typically involve the collection of multiple data sets on the same crystal as these conditions are varied. To obtain the most statistically significant insight into such changes, a large collection of data points is preferred. Additionally, pinpointing the precise condition for any structural change is a valuable characterization. This also may require collection of a large number of data sets. As a result, these types of experiments have traditionally been limited by data collection times.

**Figure 1:**
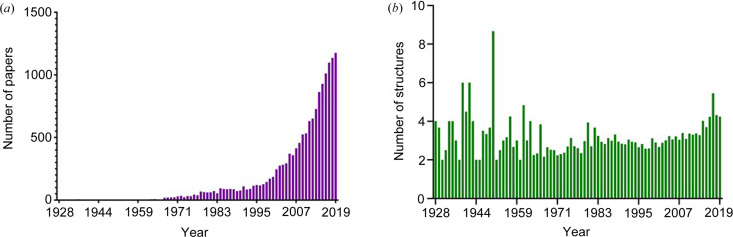
(*a*) Number of papers per year that include multiple structures of the same compound. (*b*) For the papers that publish multiple structures of the same compound, the average number of structures in a single paper is plotted against the year. For both of these graphs, ‘the same compound’ is defined as those having an identical prefix in their refcode within the CSD (*i.e* ACACCU, ACACCU01, ACACCU02 *etc.*).

Faster collection times on home sources are now making these experiments more common. For example, one recent investigation into a spin cross-over material yielded more than 200 crystal structures, with each individual data collection requiring an average of only 20 minutes for a complete data set (Zuluaga *et al.*, 2020 ▸). Depositions in the Cambridge Structural Database (CSD; Groom *et al.*, 2016 ▸) show that the number of papers including multiple crystal structures of the same material is increasing exponentially [Fig. 1 ▸(*a*)]. Though instrument technology has advanced, the software required to handle such large data sets has not progressed to the same extent, as highlighted by the average number of structures in such papers not yet demonstrating significant growth [Fig. 1 ▸(*b*)]. The time required to manually model large collections of data sets is not inconsequential; considering many of the structures may be near identical, it is not an efficient use of time for any researcher. Clearly, for dynamic crystallographic experiments to become widely viable, simple and freely available data processing analysis software is required to complement the current state of hardware development.

### 1.2. Reference structures for multiple refinements

Our approach to automating the analysis of serial experiments is the use of a reference structure. For cases where the only difference in the crystal structure is a subtle change of the unit cell axis, the same model can be refined independently against the reflection files from all data sets collected. This eliminates the requirement for manual model building and instead allows for time-saving automation. Many dynamic experiments, however, also include changes of symmetry or the extent of disorder within the structure. In such cases, one reference structure is not appropriate, but this is easily solved by utilizing multiple reference structures. For example, this could be a different reference for each crystallographic phase, or one model with disorder and one without. Refining all output reflection files from a serial collection against multiple references allows for automation to still be executed to account for variations across data sets. This also highlights the potential to compare multiple models quickly and efficiently across a series of data sets to identify points of interest or allow the crystallographer to decide which model best suits each data set. Given that significant work has already been undertaken in the development of auto-processing software for both home sources (Agilent, 2014 ▸) and synchrotrons (Winter *et al.*, 2018 ▸), our efforts in automating dynamic experiments are initially focused on post-data-reduction analysis, where refinement using reference structures is a key aspect for automation.

## 2. CX-ASAP

**Figure 2:**
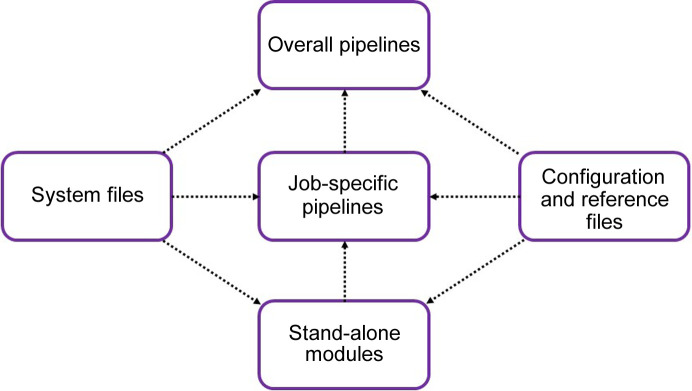
Hierarchy of the modular architecture of *CX-ASAP*. Modules perform single tasks on a single structure. They feed into job-specific pipelines which execute the module over a series of structures. In turn, this feeds into overall pipelines which execute multiple job-specific pipelines. System files and configuration files are fed into all types. Flowcharts for the refinement module and the general pipeline are given in Figs. S1 and S2 of the supporting information, with additional flowcharts for the remaining modules and pipelines available on the *CX-ASAP* website (https://cx-asap.github.io/FLOWCHART.html)

*CX-ASAP* (*C* *hemical Xtallography – Australian Synchrotron Auto-Processing*) is a modular operating-system-independent software package written in Python3, designed to increase the throughput of data processing and analysis for dynamic crystallographic experiments. The architecture of this software can be broken down into three key categories: configuration files, modules and pipelines. The configuration files provide modules and pipelines with information about the operating system, processing requirements and reference structures. Modules perform various tasks, such as structure refinement on individual data sets, and are discussed in Section 2.1. Pipelines call these modules either to execute them for a series of individual data sets (job-specific pipelines) or to run various tasks over a series of data sets (overall pipelines). Software dependencies are given in the supporting information. These job-specific pipelines feed into overall pipelines, providing the code with a hierarchical architecture (Fig. 2 ▸). There are several advantages of this kind of modular architecture: the code is more robust, the experiments can be tailored and the capabilities of the software package can be readily expanded.

The focus of *CX-ASAP* version 1.0.0 is post-data-reduction analysis (although later releases incorporating data reduction are planned). It requires a series of reflection/*SHELX* instruction files (.hkl/.ins), along with a single reference structure (.cif, .ins or .res). For cases where multiple reference structures are required, the code would be executed multiple times, each time declaring a different reference structure. Automating this process of using multiple reference structures is also planned for future releases. Utilizing the full capabilities of *CX-ASAP* version 1.0.0 will allow for full refinement of structures based on the reference, finalization of CIFs and analysis of the output. The modular design, however, means that steps may be run individually or be skipped entirely. For example, if the modelling of a dynamic experiment proved to be problematic, where every structure was in a different space group, or had greatly varying disorder models, the refinements may require manual intervention, but the CIF finalization and analysis modules could still be run to provide quick examination of the results. The other key advantage of the modular design is that different pipelines can be scripted for customized file structures/instrument output systems. This means that the user is required to configure fewer components manually. The advantage of making the code open source is that different users will be able to tailor their own pipelines for their own in-house configurations.

### 2.1. Modules

#### 2.1.1. Refinement module

The refinement module handles the tasks of importing reference structures and executing the *SHELXL* program (Sheldrick, 2015 ▸) to refine a reflection file against the reference structure. The reference structure is defined as all *SHELX* commands from LATT to END. This includes the symmetry requirements, any constraints or restraints, additional commands, and the fractional coordinates of the asymmetric unit cell. Essentially, this section comprises the entire file except for the dimensions of the unit cell (excluding TITL, CELL and ZERR). The reference information is then combined with the relevant cell information to form a new instruction file and *SHELXL* is executed via the command line. For a structure to be considered fully refined, it is expected that the weighting scheme is unchanged between refinement cycles and the shift of model parameters is zero. The refinement module was designed to automatically refine to completion, with some exceptions allowing for user tailoring.

**Figure 3:**
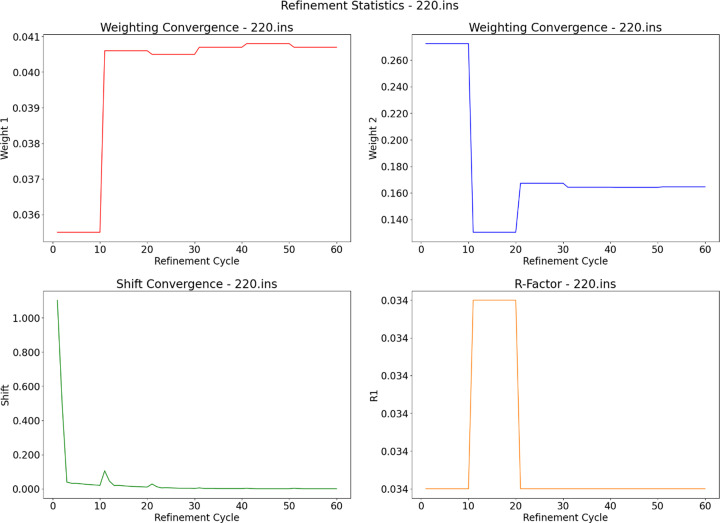
Example output graphs from the refinement module using a test data set included in the *CX-ASAP* package. They show how various statistics change during the refinement cycles. Top left: first number in the weighting scheme. Top right: second number in the weighting scheme. Bottom left: shift value. Bottom right: *R*_1_ factor.

Convergence is defined by two criteria: the weighting scheme must be unchanging and the average shift of the most recent ‘X’ cycles must be below a tolerance of ‘Y’, which are parameters that can be changed by the user. The default convergence criteria request that the most recent eight cycles have an average shift below 0.002 (‘X’ = 8 and ‘Y’ = 0.002). The code was structured in this way to allow for the option of ‘quick and dirty’ refinements to quickly check data quality prior to committing computer resources to long refinements (particularly important for structures with large unit cells). Only a set number of cycles are averaged to avoid the effect of outliers, as the first cycle will always have a significantly higher shift value. *SHELXL* will continue to be called until convergence is reached, or a maximum number of executions have been run (user-defined limit with a default of 20 runs). Once convergence or the maximum number of permitted cycles has been reached, graphical output assists the user in determining whether their refinement is suitable or not (Fig. 3 ▸).

Each output consists of four graphs describing how various statistics changed throughout the refinement. The top two graphs show how the weighting scheme converged. As this is typically represented by two numbers in *SHELXL*, it has been displayed over two graphs. The top two graphs clearly show some refinement of the weighting scheme, which appears to have converged. The bottom-left graph shows the shift values converging and the bottom-right graph shows how the *R* factor changes during refinement. This module also keeps track of which structures have been successfully refined, so when it is used over multiple data sets the user can quickly see which structures worked and if any failed. When executed over a series of data sets in a job-specific pipeline, the software will output a text file summarizing which data sets successfully refined and which (if any) failed.

#### 2.1.2. CIF finalization module

With the automatic refinement of data sets and the capability of *SHELXL* to automatically write the CIF on completion, it was a natural extension of *CX-ASAP* to finalize these files as well. The initial CIF written by *SHELXL* contains the required structure information but lacks details about the diffractometer and various parameters used during data collection. These can be provided in the form of a reference CIF, from which the code can extract the instrumentation information, assuming it was the same for all data sets. Alternatively, there is also the capability for the code to merge the structure CIF with an instrument CIF tailored for each data set, with the user able to define additional parameters such as crystal size, colour, habit *etc*. Ultimately, the quality of the output files is entirely defined by the quality of the crystallographer input. If a high-quality, complete reference is used and nothing unexpected occurs during data refinement, then the resulting CIFs could be ready for publication, although they should always be checked carefully by the user prior to doing so. *CX-ASAP* provides the tools to do this, by generating an automatic *checkCIF* report using the software package *PLATON* (Spek, 2020 ▸), as well as graphical depictions of the structure quality statistics. Ultimately, even in cases where the CIFs are not ready for publication, they provide an ideal medium for quick comparison via the analysis modules, which easily extract information for comparison across data sets.

#### 2.1.3. Analysis modules

**Figure 4:**
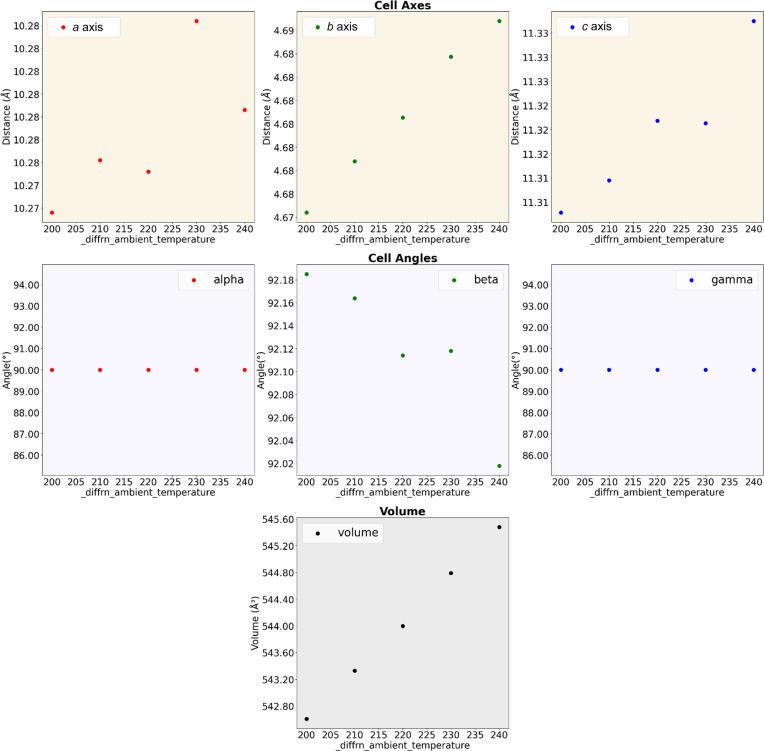
Example graphical output for the unit-cell deformation using the test data set included in the *CX-ASAP* package. Fast examination of the cell parameters quickly identifies potential issues in the data quality. For instance, in this case the data do not have clear trends for several of the parameters (*a* axis, *b* axis, β), indicating that further investigation is required.

A series of analysis modules is included within the *CX-ASAP* package to provide fast visualization and analysis of large data sets. This is critical to ensure that any discrepancies or properties are not overlooked. For instance, a structure refined in the incorrect symmetry setting will generally display worse quality statistics or a change in trend to assist with identification. The analysis modules help with ascertaining the success of the experiment by extracting the desired parameters from the output CIFs, providing them in a convenient spreadsheet format (.csv file) and displaying them in graphical format. Usually, the cell parameters, quality statistics and structural information are extracted, but these can be tailored by the user to pull out any desired parameter. Some of the graphs included show how the unit cell changes (Fig. 4 ▸), or how the statistics change across the experiment.

### 2.2. General post-data-reduction pipeline

The combination of these modules results in a general post-data-reduction pipeline. When provided with a series of .ins/.hkl files within separate folders, as well as a reference CIF, *CX-ASAP* will run the modules in the required order to perform fully automated finalization of the data. For cases where the user does not already have their data in the correct file structure, the file tree can be set up for the user using additional optional steps. This is also the case for users who may not have a reference CIF, but rather provide the required instrumentation details manually for finalization. Note that there is currently no way to automatically generate a reference structure, as this has the potential of introducing significant errors into the output. A well refined, manually generated reference structure remains the best way to aim for publication-quality output. Though this general pipeline is the only overall pipeline included in this initial release of *CX-ASAP*, the modular design means that other pipelines can be configured for specific applications to increase the level of automation. For example, other pipelines under development include those that take the output of files from the current Australian Synchrotron auto-processing pipeline to automate the entire process on macromolecular crystallography beamlines (Aragão *et al.*, 2018 ▸, Cowieson *et al.*, 2015 ▸). Others are also under active development for home-source diffractometers, which will likely be the most common use for this software.

### 2.3. Experiment configuration

*CX-ASAP* operates through a command line interface. This interface guides the user through running the code with step-by-step instructions. Users can opt to run individual modules, job-specific pipelines or overall pipelines. Once the desired procedure has been selected, and the user has been informed of the software/file dependencies, *CX-ASAP* requires configuration. This is carried out by the user editing a file called conf.yaml. This dictionary-type file allows for parameters such as the location of the user data to be specified, alongside other options required for the various modules. Exactly what is required for each module/pipeline is explained through the command-line interface. The command-line interface also contains a ‘test’ function to ensure all software dependencies are installed correctly and an ‘errors’ command to display the current error log for troubleshooting.

## 3. Application of *CX-ASAP*

A recent study investigating the spin-crossover behaviour of an Fe(III) complex co-crystallized with 1,3,5-tri­iodo­tri­fluoro­benzene demonstrated unique spin-crossover switching behaviour driven by single-crystal-to-single-crystal phase transitions on solvation/desolvation (Zuluaga *et al.*, 2020 ▸). One of the methods used to investigate this behaviour was a range of variable-temperature single-crystal diffraction experiments. In one variable-temperature experiment, 64 individual data sets were collected using the strategy in Table 1 ▸. The authors indicated that they required approximately 400 working hours to manually perform the full refinement and finalization of these structures. The combined CIF for this study (1·IFB·MeOH) was downloaded from the CSD and run through *CX-ASAP*.

As described in the publication, desolvation occurs above 350 K. As such, the individual CIFs for the first and last data sets were extracted to cover both solvated and desolvated structures. *CX-ASAP* was run for both reference structures individually. In doing so, many of the structures failed to refine against either reference. On quick inspection, we found that some of the structures were in different settings of the triclinic unit cell. Two additional reference CIFs (solvated and desolvated) in the alternative unit-cell setting were chosen, and the code was run an additional two times using the new references. Overall, the entire pipeline was executed four times, one for each reference structure, and the outputs were compared to see which reference was required for each data set to successfully refine. At this point, all structures were successfully refined, with the only *checkCIF* alerts present matching the kinds of alerts present in the original structures. This took approximately 45 min of computation time (see supporting information for hardware used) and only 15 min of manual time to configure the code and identify the need for new references. Not only is this substantially faster, but it also highlights the power of the code to quickly identify problems across multiple data sets.

**Figure 5:**
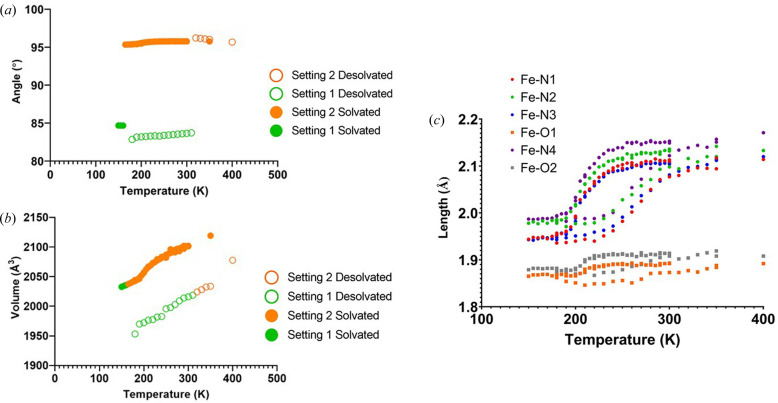
(*a*) Change in the α angle over the variable-temperature experiment. The two different settings are clearly visualized because of the large changes between solvated and desolvated cell settings. (*b*) Change in unit-cell volume over the variable-temperature experiment, demonstrating the hysteresis and spin-crossover behaviour. (*c*) Changes in bond lengths within the Fe(III) coordination sphere which further highlight the spin-crossover behaviour.

Owing to the range of references involved as the unit cells were in different settings, the data required manual combination to graph the changes in unit-cell parameters. This was fast and efficient due to the output of these data in .csv format. From here, the data sets could be quickly copied and pasted into a new spreadsheet to visualize the unit-cell trends. The bond lengths within the Fe(III) coordination sphere, key indicators of the spin state, were also automatically extracted from the CIFs and graphed similarly (Fig. 5 ▸).

## 4. Conclusions and installation

The collection of multiple data sets on dynamic systems is important to probe material characterization and fundamental crystallographic understanding. *CX-ASAP* is a new open-source tool to analyse these experiments efficiently. The use of a publication-standard reference structure empowers scientists to automatically refine multiple data sets from dynamic experiments to completion and quickly identify problematic areas. The code can be downloaded from GitHub (https://github.com/cx-asap/CX-ASAP) and the current release runs on Linux, OSX and Windows, although many new features under development require a Unix-style architecture. Installation instructions are contained within the README.md text file contained within the downloaded package along with a list of software dependencies. A test data set (Brock *et al.*, 2018 ▸) is also included to troubleshoot installation requirements. This software package has been successfully deployed to quickly investigate crystalline properties (Bhandary *et al.*, 2020 ▸; Thompson, Price *et al.*, 2021 ▸), and it is envisioned that wider deployment will assist with in-depth understanding of crystalline properties.

## Supplementary Material

Supporting figures and tables. DOI: 10.1107/S1600576723000298/yr5097sup1.pdf

## Figures and Tables

**Table 1:** Variable-temperature collection strategy employed to investigate single-crystal-to-single-crystal phase transitions (Zuluaga *et al.*, 2020 ▸)

Temperature range (K)	Step size (K)
150–300	5
290–180	10
300–400	50
350–180	10

Total No. of structures	64

## References

[bb1] Aalst, W. M. P. van der, Bichler, M. & Heinzl, A. (2017). *Bus. Inf. Syst. Eng.* **59**, 311–313.

[bb2] Agilent (2014). *CrysAlis PRO.* Agilent Technologies Inc., Santa Clara, CA, USA.

[bb3] Aragão, D., Aishima, J., Cherukuvada, H., Clarken, R., Clift, M., Cowieson, N. P., Ericsson, D. J., Gee, C. L., Macedo, S., Mudie, N., Panjikar, S., Price, J. R., Riboldi-Tunnicliffe, A., Rostan, R., Williamson, R. & Caradoc-Davies, T. T. (2018). *J. Synchrotron Rad.* **25**, 885–891. 10.1107/S1600577518003120 PMC5929359 29714201

[bb4] Bhandary, S., Thompson, A. J., McMurtrie, J. C., Clegg, J. K., Ghosh, P., Mangalampalli, S., Takamizawa, S. & Chopra, D. (2020). *Chem. Commun.* **56**, 12841–12844. 10.1039/d0cc05904h 32968742

[bb5] Brock, A. J., Whittaker, J. J., Powell, J. A., Pfrunder, M. C., Grosjean, A., Parsons, S., McMurtrie, J. C. & Clegg, J. K. (2018). *Angew. Chem. Int. Ed.* **57**, 11325–11328. 10.1002/anie.201806431 29998602

[bb6] Bushuyev, O. S., Corkery, T. C., Barrett, C. J. & Friščić, T. (2014). *Chem. Sci.* **5**, 3158–3164.

[bb7] Cowieson, N. P., Aragao, D., Clift, M., Ericsson, D. J., Gee, C., Harrop, S. J., Mudie, N., Panjikar, S., Price, J. R., Riboldi-Tunnicliffe, A., Williamson, R. & Caradoc-Davies, T. (2015). *J. Synchrotron Rad.* **22**, 187–190. 10.1107/S1600577514021717 PMC4294030 25537608

[bb8] Groom, C. R., Bruno, I. J., Lightfoot, M. P. & Ward, S. C. (2016). *Acta Cryst.* B**72**, 171–179. 10.1107/S2052520616003954 PMC4822653 27048719

[bb9] Helliwell, J. R. (2019). *Struct. Dyn.* **6**, 054306. 10.1063/1.5124439 PMC6816445 31673568

[bb10] Moggach, S. A., Bennett, T. D. & Cheetham, A. K. (2009). *Angew. Chem. Int. Ed.* **48**, 7087–7089. 10.1002/anie.200902643 19681088

[bb11] Sheldrick, G. M. (2015). *Acta Cryst.* C**71**, 3–8.

[bb12] Spek, A. L. (2020). *Acta Cryst.* E**76**, 1–11. 10.1107/S2056989019016244 PMC6944088 31921444

[bb13] Thompson, A. J., Price, J. R., McMurtrie, J. & Clegg, J. K. (2021). *Nat. Commun.* **12**, 5983. 10.1038/s41467-021-26204-z PMC8528856 34671030

[bb14] Thompson, A. J., Worthy, A., Grosjean, A., Price, J. R., McMurtrie, J. C. & Clegg, J. K. (2021). *CrystEngComm*, **23**, 5731–5737.

[bb15] Wilkinson, M. D., Dumontier, M., Aalbersberg, I. J., Appleton, G., Axton, M., Baak, A., Blomberg, N., Boiten, J. W., da Silva Santos, L. B., Bourne, P. E., Bouwman, J., Brookes, A. J., Clark, T., Crosas, M., Dillo, I., Dumon, O., Edmunds, S., Evelo, C. T., Finkers, R., Gonzalez-Beltran, A., Gray, A. J., Groth, P., Goble, C., Grethe, J. S., Heringa, J., ’t Hoen, P. A., Hooft, R., Kuhn, T., Kok, R., Kok, J., Lusher, S. J., Martone, M. E., Mons, A., Packer, A. L., Persson, B., Rocca-Serra, P., Roos, M., van Schaik, R., Sansone, S. A., Schultes, E., Sengstag, T., Slater, T., Strawn, G., Swertz, M. A., Thompson, M., van der Lei, J., van Mulligen, E., Velterop, J., Waagmeester, A., Wittenburg, P., Wolstencroft, K., Zhao, J. & Mons, B. (2016). *Sci. Data* **3**, 160018.

[bb16] Winter, G., Waterman, D. G., Parkhurst, J. M., Brewster, A. S., Gildea, R. J., Gerstel, M., Fuentes-Montero, L., Vollmar, M., Michels-Clark, T., Young, I. D., Sauter, N. K. & Evans, G. (2018). *Acta Cryst.* D**74**, 85–97. 10.1107/S2059798317017235 PMC5947772 29533234

[bb17] Zuluaga, A. R., Brock, A. J., Pfrunder, M. C., Phonsri, W., Murray, K. S., Harding, P., Micallef, A. S., Mullen, K. M., Clegg, J. K., Harding, D. J. & McMurtrie, J. C. (2020). *Chem. Mater.* **32**, 10076–10083.

