# Supplementary material for: CX-ASAP: a high-throughput tool for the serial refinement and analysis of crystallographic data collected under varying conditions
Source: J Appl Crystallogr. 2023 Feb 28;56(Pt 2):558–64. doi: 10.1107/S1600576723000298 (PMC10077850; doi:10.1107/S1600576723000298)
Supplement: Supplementary file 1 [file j-56-00558-sup1.pdf]

JOURNAL OF
APPLIED
CRYSTALLOGRAPHY

Volume 56 (2023)

Supporting information for article:

CX-ASAP: A high throughput tool for the serial refinement and analysis of crystallographic data collected under varying conditions

Amy J. Thompson, Kate M. L. Smith, Jack K. Clegg and Jason R. Price

For installation instructions and contribution guidelines please head to our website (https://cx-asap.github.io/). The code is housed at the following repository: https://github.com/cx-asap/CX-ASAP

S1. Dependencies

CX-ASAP has not been exhaustively tested with a range of software versions. Therefore, it is recommended to set up an environment following the tested software versions in Table S1. Dependencies for additional python packages are included in the *requirements.txt* file and will be automatically installed upon installation of *CX-ASAP*. Again, it is recommended that it is installed in an environment to avoid clashes between software dependencies and to ensure smooth installation.

Table S1 Tested dependencies for *CX-ASAP* version 1.0.0

**Software**	**Windows**	**MacOSX**	**Linux**
Python	3.6 - 3.8	3.6 – 3.8	3.6 – 3.8
SHELXL	2018/3	2018/3	2018/3
ShredCIF	2014/3	2014/3	2014/3
PLATON	070722	210622	140520

S2. Hardware for Benchmarking

The automatic analysis run performed in section **3. Application of CX-ASAP** used a laptop computer with the hardware listed in Table S2.

Table S2 Hardware used for benchmarking the performance of *CX-ASAP* in section **3. Application of CX-ASAP**

**Property**	**Hardware**
Architecture	x86_64
CPU	Intel® Core™ i7-9750H CPU @ 2.60 GHz
CPUs	12
Threads per core	2
Cores per socket	6
RAM	32 GB
Operating System	Linux Mint 20 (Ulyana)

S3. Available Modules and Pipelines

The modules and pipelines currently available within *CX-ASAP* version 1.0.0 are detailed below. All modules are run for a single structure, while all job-specific pipelines simply extend modules to run over a series of structures. Overall pipelines execute job-specific pipelines sequentially according to the crystallographic pipeline. Flowcharts highlighting the

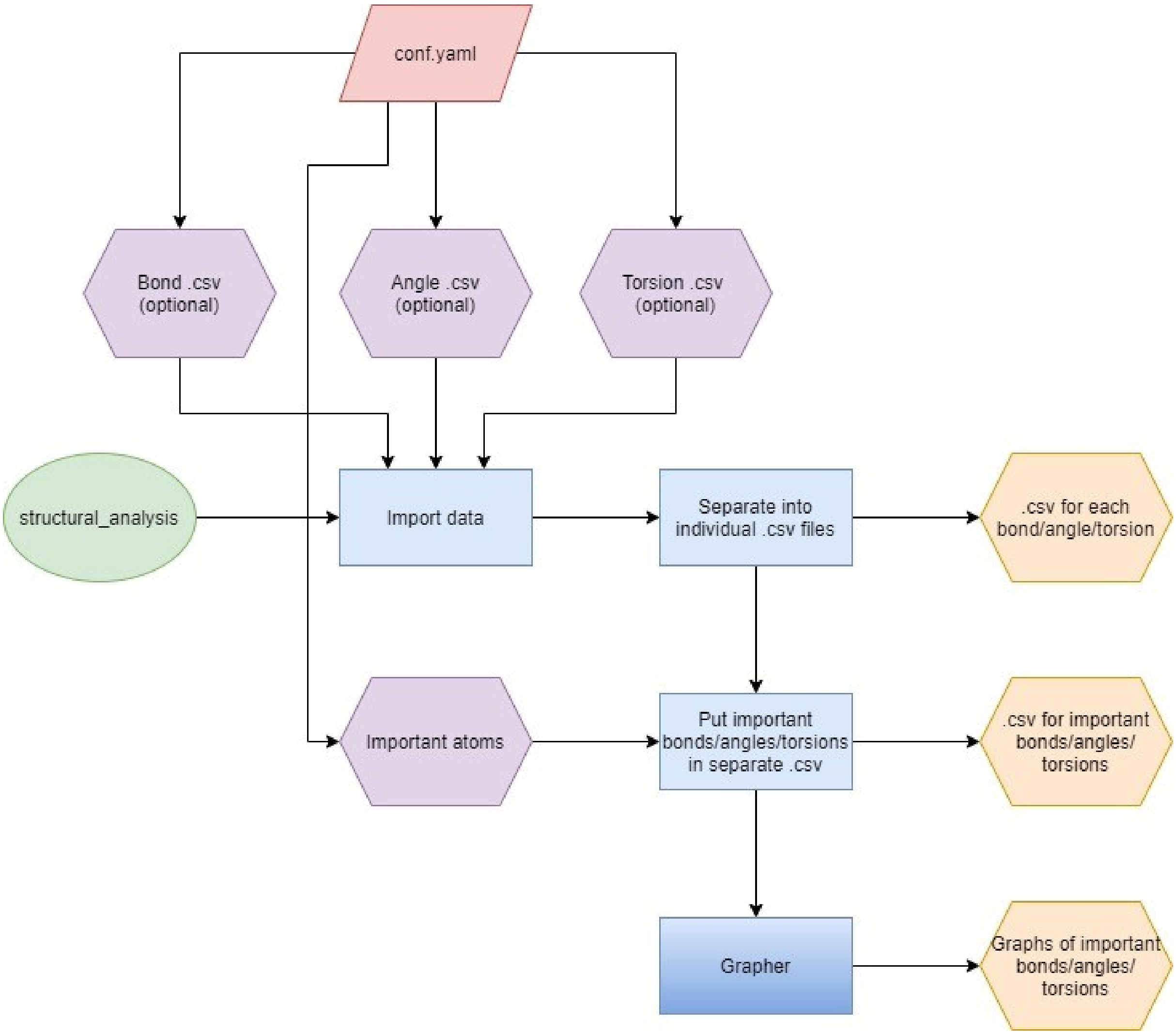

Figure S1 Flowchart for the workflow of the refinement module. Flowcharts for further modules and pipelines are located at: https://cx-asap.github.io/FLOWCHART.html

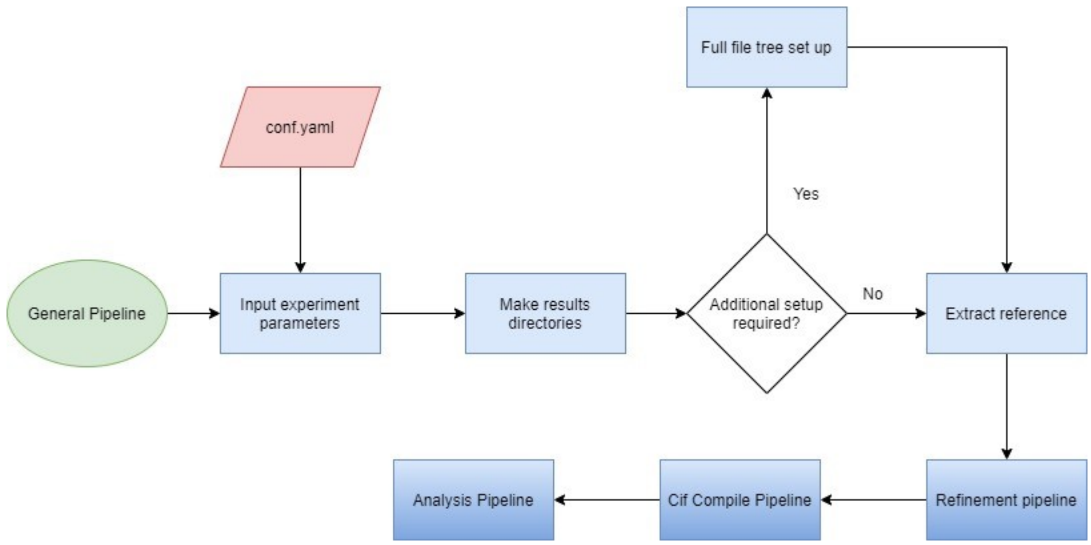

Figure S2 Flowchart for the general pipeline. Flowcharts for further modules and pipelines are located at: https://cx-asap.github.io/FLOWCHART.html

Table S3 List of modules available in *CX-ASAP* version 1.0.0.

**Stage in Crystallographic Pipeline**	**Available Modules**	**Description**
Data Refinement	Refinement	Executes SHELXL on a single structure and checks for convergence
CIF Validation	CIF Merge	Includes instrument parameters, runs checkCIF through PLATON and merges output
	Instrument CIF Generation	Generates a CIF with instrument information from a fully refined structure CIF
Post Refinement Analysis	ADP Analysis	Analyses the ADP parameters of atoms
	Cell Analysis	Analyses/graphs changes in the unit cell
	CIF Read	Extracts key information from refined CIF files
	Rotation Planes	Analyses change in angle between defined plane and those used in the MPLA SHELX command
	Structural Analysis	Graphs changes in bond lengths, angles and torsions

Table S4 List of pipelines available in *CX-ASAP* version 1.0.0.

Type of Pipeline	Stage in Crystallographic Pipeline	List of Pipelines in CX-ASAP	Description
Job-Specific	Data Refinement	Refinement Pipeline	Refines a series of structures
	CIF Validation	CIF Combine	Combines CIFs ONLY
		CIF Pipeline	Combines CIFs, runs checkCIF, and merges instrument data
	Post Refinement Analysis	Rotation Pipeline	Looks at changes in rotation angle over a series of structures
		Variable CIF Parameter	Analyses changes in structural parameters based on a defined parameter in the output CIFs changes (ie _diffrn_ambient_temperature)
Overall Pipelines	Data Refinement, CIF Validation, Post Refinement Analysis	CX-ASAP Pipeline	Combines data refinement, CIF validation and post refinement analysis into a single pipeline

S4. Additional Modules Planned for Future Release

The below tables include code that is in active development for future release of *CX-ASAP*. These will be available on the GitHub page as a development branch (v 1.1.0) separate from the main branch. These modules have not been benchmarked or fully tested across a variety of systems and thus carry an increase likelihood of bugs and errors. Some modules also rely on software that requires a unix architecture (XDS) and proprietary (XPREP).

Table S5 Modules in active development for future releases of *CX-ASAP*.

**Stage in Crystallographic Pipeline**	**Available Modules**	**Description**
Data Reduction	XDS Cell Transformation	Applies a defined unit cell transformation to XDS data
	XDS Reprocess	Runs XDS on a single structure
	XPREP Intensity Compare	Analyse how reflection conditions change between two datasets
	XPREP Module	Runs XPREP on a single structure
Additional Tools	Molecule reconstruction and extrapolation	Alters unit cell and reconstructs molecule in theoretical unit cell
	SHELXT	Automatic SHELXT for a single structure
	PLATON Squeeze	Apply PLATON's squeeze function

Table S6 Modules in active development for future releases of *CX-ASAP*.

**Type of Pipeline**	**Stage in Crystallographic Pipeline**	**List of Pipelines in CX-ASAP**	**Description**
Job-Specific	Data Reduction	XDS Pipeline	Refines a series of structures
		XPREP Intensity Pipeline	Combines CIFs ONLY
		XPREP Pipeline	Combines CIFs, runs checkCIF, and merges instrument data
	Post Refinement Analysis	Variable Position Analysis	Analyses changes specific for an experiment where the position across the crystal changes
		Variable Temperature Analysis	Analyses changes specific for an experiment where the temperature changes
	Additional Tools	SHELXT Pipeline	Applies SHELXT to a series of structures
		PLATON SQUEEZE Pipeline	Applies PLATON SQUEEZE to a series of structures
Overall Pipelines	Data Refinement, CIF Validation, Post Refinement Analysis	Rigaku Synergy VT Pipeline	Automates analysis of variable temperature experiments run on a Rigaku instrument
	Data Reduction, Data Refinement, CIF Validation, Post Refinement Analysis	Variable Position Pipeline	Analyses variable position experiments
	Data Refinement, CIF Validation, Post Refinement Analysis	Variable Temperature Pipeline (AS)	Analyses variable temperature experiments run at the Australian Synchrotron
